# Dr. Cral Von Arx Schneider

**Published:** 1917-03

**Authors:** 


**Dr. Cral von Arx Schneider,** Cleveland Homoeopathic 1904, Assistant Supt. of the Gowanda State Hospital, died at that institution Jan. 29 of typhoid fever.

